# Effect of Standardized Warfarin Treatment Protocol on Anticoagulant Effect: Comparison of a Warfarin Medication Therapy Adherence Clinic with Usual Medical Care

**DOI:** 10.3389/fphar.2017.00637

**Published:** 2017-11-09

**Authors:** Salihah Aidit, Yee Chang Soh, Chuan Seng Yap, Tahir M. Khan, Chin Fen Neoh, Shazwani Shaharuddin, Yaman W. Kassab, Rahul P. Patel, Long C. Ming

**Affiliations:** ^1^Faculty of Pharmacy, Universiti Teknologi MARA, Puncak Alam, Malaysia; ^2^Faculty of Pharmaceutical Sciences, UCSI University, Kuala Lumpur, Malaysia; ^3^School of Pharmacy, Monash University, Sunway City, Malaysia; ^4^Collaborative Drug Discovery Research Group, Pharmaceutical and Life Sciences Communities of Research, Universiti Teknologi MARA, Shah Alam, Malaysia; ^5^Department of Hospital and Clinical Pharmacy, Faculty of Pharmacy, Cyberjaya University College of Medical Sciences, Cyberjaya, Malaysia; ^6^Pharmacy, School of Medicine, University of Tasmania, Hobart, TAS, Australia; ^7^School of Pharmacy, KPJ Healthcare University College, Nilai, Malaysia

**Keywords:** warfarin, anticoagulant, international normalized ratio, percent time in therapeutic INR range, multidisciplinary care

## Abstract

**Objective:** To evaluate the impact of pharmacist-led warfarin management and standardized treatment protocol.

**Methods:** A retrospective cohort study was carried out in a cardiology referral hospital located in central Kuala Lumpur, Malaysia, from 2009 to 2014. The inclusion criteria were: adult patients who were diagnosed and treated for atrial fibrillation (AF) with warfarin, attended the warfarin medication therapy adherence clinic (WMTAC) for at least 12 weeks, and with at least four international normalized ratio (INR) readings. The electronic medical records were reviewed for demographics, type of AF, warfarin dose, INRs, adverse events, co-morbidities, and drug–drug interactions. The outcome measures included the mean time to therapeutic INR, the mean percentage of time in therapeutic range (TTR), bleeding events, and common drug interactions.

**Results:** Out of 473 patients, 151 patients fulfilled the inclusion criteria. The findings revealed that there were significant associations between the usual medical care (UMC) group and pharmacist-led WMTAC in terms of TTR (*p* = 0.01) and INR (*p* = 0.02) levels. A positive impact of pharmacists’ involvement in the WMTAC clinic was where the “pharmacist’s recommendation accepted” (*p* = 0.01) and “expanded therapeutic INR range” (*p* = 0.04) were statistically significantly higher in the WMTAC group.

**Conclusion:** There was a significant positive association between the pharmacist-led WMTAC and anticoagulation effect (therapeutic TTR, INR). The identified findings revealed that expanded role of pharmacist in pharmacist-managed warfarin therapy is beneficial to optimize the warfarin therapy. This study also highlighted the critical roles that pharmacists can actively play to ensure optimal anticoagulation pharmaceutical care.

**Key messages:**

**What is already known on this subject?**

• Pharmacist-managed warfarin therapy is beneficial for optimizing warfarin therapy. In such therapy, recommendations such as dose adjustment and safer alternative drugs (given drug–drug interactions and/or food–drug interactions) are made.
• The active involvement of pharmacists in warfarin adherence clinics could significantly improve adherence.
• However, the warfarin treatment outcomes from UMC, pharmacist-and-physician-led care and pharmacist-led care have not been studied.
• The impact of the implementation of the standardized protocol for the warfarin adherence clinic has not been assessed.

**What this study adds?**

• INR levels among UMC group and WMTAC group were significantly different.
• Though the TTR level for the WMTAC group was not significantly different than the UMC group, it was higher and close to the targeted 60% level.
• The identified findings show that pharmacists’ focus on intervention for missed doses, adherence and dose adjustment provide positive impact on patients’ warfarin therapy.

## Introduction

Atrial fibrillation (AF) constitutes a significant public health problem and is considered the most common arrhythmia of clinical significance (Zubaid et al., 2013). Due to the growing prevalence and incidence of AF across the world, recent epidemiological statistics confirm the emergence of this disorder as a global epidemic (Ministry of Health Malaysia, 2012). In 2010, it was estimated that about 33.5 million individuals, or 0.5% of the world’s population, has AF (Zubaid et al., 2013). This rise in the epidemiology of AF is expected to continue with the aging of societies worldwide (La Brooy and Ho, 2015).

Although frequently associated with palpitations and fluttering, AF remains asymptomatic for many patients. One of the main risk factors of AF is hypertension, and a study by Wong et al. (2013) reported that 0.75% of hypertensive patients have asymptomatic AF (AAF). The risk is similar among both genders. Asymptomatic patients with comorbid hypertension aged 61 and above were associated with a 10.6 times higher probability of AAF. Wong et al. (2013) estimated that there were 49,029 Malaysians with AAF in 2010. Hence, greater emphasis on diagnostic ascertainment, screening, and prevention strategies are important to reduce the risk of AAF-related complications (Wong et al., 2013).

Asian populations were reported to have lower incidence and prevalence of AF than Western populations. The estimate is 0.4–1% of the general Asian population. However, the relative risks of AF-associated stroke and mortality in Asian and Western populations are similar (Ministry of Health Malaysia, 2012; La Brooy and Ho, 2015). According to guidelines for management of AF in United States and Europe, non-valvular AF patients with additional risk factors for ischemic stroke and systemic thromboembolism should be prescribed with chronic oral anticoagulants, of which warfarin remains the gold standard. Both local and international literature have pointed out the lack of proper standard treatment guidelines and recommendations regarding how to manage patients taking warfarin. The treatment is often complicated when patients are on dual antiplatelet therapy. Patients discharged from hospital with concomitant aspirin, clopidogrel, and warfarin are subjected to greater risk of bleeding events. Furthermore, patients’ outcomes can be affected by interactions of warfarin with food or traditional and complementary medicines.

To the best of our knowledge, only one study so far has been conducted in Malaysia to evaluate pharmacists’ management of warfarin treatment, but this particular study did not evaluate the impact of the warfarin medication therapy adherence clinic (WMTAC) protocol, which was implemented in January 2013 (Thanimalai et al., 2013).

The main aim of the study was to compare the international normalized ratio (INR) and percentage of time in therapeutic range (TTR) levels among a group of AF patients who received warfarin under usual medical care (UMC) before the Malaysian warfarin protocol was implemented, and among group of AF patients who received warfarin under the WMTAC after the implementation of the protocol. This study also aimed to determine the therapeutic outcomes (for example, the number of bleeding events) and interventions conducted by the WMTAC pharmacists.

## Materials and Methods

### Study Setting

This study was a retrospective observational study conducted at a public hospital located in Kuala Lumpur, Malaysia.

### Study Design

**FIGURE 1:**
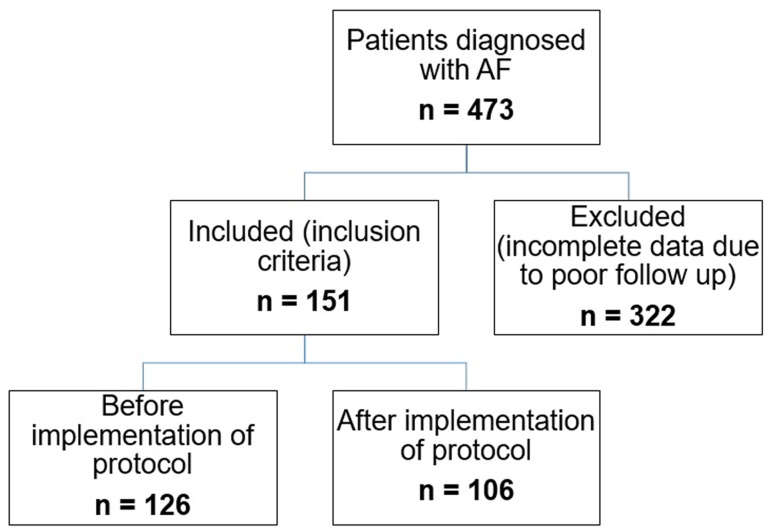
Sampling of patients.

This was a retrospective cohort study using pre- versus post-WMTAC protocol design. AF patients who followed up in the warfarin clinics between 2009 and 2014 were considered and their medical records were retrieved. The flow chart for recruitment of patients is shown in **Figure 1**.

This study consisted of two groups:

(1) Group 1 (pre-protocol group): patients were recruited from January 2009 until December 2012. This group consisted of patients who were initiated on warfarin under standard medical team management known as UMC before the WMTAC protocol was introduced. The warfarin clinic was mainly led by physicians and a referral to a pharmacist was only made when necessary.
(2) Group 2 (post-protocol group): patients were recruited from January 2013 until December 2014, after implementation of the new Ministry of Health (MOH) WMTAC protocol. This group was managed by both pharmacists and physicians and called the WMTAC group. In this group, pharmacists were more involved, and have expanded role in patient education and counseling. They were also allowed to implement the protocol and recommend any dosage adjustment and/or continuation of warfarin therapy.

### Ethics Statement

The study protocol was approved by the Clinical Research Centre of the patients’ recruitment hospital and the Medical Research & Ethics Committee at the Malaysian Ministry of Health. All data collection and information was kept confidential according to the ethical requirements. All aspects of the study protocol, including access to and use of the patient clinical information was authorized by the medical ethics committee and the local health authorities before initiation of the study. Prior approval to conduct this study was obtained from: Medical Research and Ethics Committee, UiTM; National Institutes of Health (NIH), Ministry of Health; and Medical Research and Ethics Committee (MREC), Ministry of Health. The NMRR registration number for this study is NMRR-14-1623-20026 (IIR).

### Study Population

#### Recruitment of Participants

All patients taking warfarin were screened initially from the hospital medical record department and the warfarin patient record book from the warfarin medical clinic. All new AF patients receiving warfarin treatment from January 2009 until December 2014 were identified and potential patients for this study were recruited using the Electronic Medical Record (EMR) system and the warfarin patient record book (**Figure 1**). The warfarin patient record book contained information such as the patient’s name, registration number, INR readings, and remarks for each patient. Patients were identified after being rechecked in the EMR.

#### Inclusion and Exclusion Criteria

All new patients who were admitted to the recruitment hospital and received warfarin during the defined study period were considered. The inclusion criteria for recruitment of patients into the study were:

(1) Adult patients (≥18 years old) who were diagnosed and treated for AF plus required warfarin therapy and attended the WMTAC clinic at the recruitment hospital.
(2) All patients diagnosed with AF and followed up regularly for at least 12 weeks with the WMTAC clinic.
(3) Stable patients on warfarin with at least four INR readings and a steady state for at least 12 weeks.

The exclusion criteria for recruitment of patients into the study were:

(1) Patients who were diagnosed with AF but had not been prescribed with warfarin.
(2) Patients who defaulted treatment or were unable to comply with the follow-up requirements.
(3) Patients who discontinued or deferred from warfarin therapy or who were discharged to another hospital at any point.
(4) Patients for whom important medical and medication data was missing.

### Data Collection

A pre-validated data collection form was used to extract clinical information on the study population from inpatient records, chart reviews, and outpatient physician office records. The information gathered was then evaluated to identify the pharmaceutical care issues following Pharmaceutical Issues Classification by Krska et al. (2002). The appropriateness of each patient’s pharmacological treatment was assessed according to local and international guidelines.

The overall percentage TTR was calculated using the method described by Rosendaal et al. (1993), which uses linear interpolation to estimate the time spent at each INR value. INR desk 4.0 Software was used to calculate the TTR percentage. An expanded INR was set between ranges of 1.8 and 3.2. The expanded INR is defined as therapeutic range INR ±0.2, and such variation of INR from therapeutic range is considered clinically insignificant, hence no dosage adjustment is required (Saokaew et al., 2012). This is in accordance with MOH WMTAC protocol (Ministry of Health Malaysia, 2010). All INR values were entered into the software and analyzed automatically for every patient. TTR readings were calculated as percentages.

### Data and Statistical Analysis

Descriptive statistics were used to describe demographic characteristics of the patients, social habits, comorbidities, CHA2DS2VASc and HASBLED scores, as well as the number of bleeding events. Percentages and frequencies were used for the categorical variables, while means and standard deviations were calculated for the continuous variables. Mann–Whitney test was used to determine the differences between interventions (expanded therapeutic INR range, missed doses, and accepted pharmacist recommendation) and INR level before and after starting the protocol. All analyses were performed using SPSS statistical software version 20 (SPSS Inc., Chicago, IL, United States). The significance level was set at *p*-value < 0.05.

### Study Outcomes

#### Primary Outcomes

The primary outcomes were the control of INR for at least 12 weeks after starting warfarin treatment and the percentage of TTR in patients in the WMTAC group compared to the UMC group.

#### Secondary Outcomes

The secondary outcome measurements included complications or adverse events (including minor bleeding symptoms) among the AF patients. Major bleeding was defined as an overt clinical bleed, or documented intracranial or retroperitoneal hemorrhage. Minor bleeding events included bruising, nose bleeds, gum bleeding, hematuria, and rectal bleeding not requiring further action. INR readings and bleeding symptoms were monitored closely at the WMTAC. A stroke was defined as an ischemic cerebral infarction caused by an embolic or thrombotic occlusion of a major intracranial artery. Examples of possible warfarin adverse events are thromboembolic and major hemorrhagic complications.

Furthermore, interventions during consultation, including pharmacists’ recommendation of dosage adjustment, expanded therapeutic INR range, and detection of missed dose, were recorded and compared for pre- and post-protocol groups.

## Results

### Characteristics of Patients

This study identified 473 AF patients who were receiving warfarin therapy. Out of the 473 patients, 62.25% (*n* = 322) patients were found ineligible as they did not fulfill the study inclusion criteria.

Patients in UMC group were selected from eligible AF patients from January 2009 until December 2012. The records of patients in WMTAC group were selected from January 2013 until December 2014. This WMTAC group consisted of new AF patients selected in the mentioned year, plus patients from the same cohort with UMC group who were still receiving warfarin therapy. In this study, 126 patients were recruited for UMC group, and 106 patients documented under WMTAC group. As some of the patients in WMTAC were continued from UMC group, the total number of patients involved were 151 subjects.

The details of the socio-demographic characteristics and common comorbidities of the patients are shown in **Table 1**. Detailed Risk Score: CHA2DS2VASc and HASBLED scores of the patients are shown in **Table 2** (note: the higher the HASBLED scores, the higher the bleeding tendency among the warfarin patients). CHA2DS2VASc and HASBLED remain as well-accepted predictive tools on risk of undesired events among warfarin patients (Lip et al., 2013).

**Table 1:** Demographic data, social habits, and comorbidities.

Demographic data and social habits		*n* (%)
Gender	Female	80 (53.0)
	Male	71 (47.0)
Race	Chinese	86 (57.0)
	Malay	59 (39.1)
	Indian	6 (4.0)
Age	30–39	3 (2.0)
[Median = 66, mean = 66.11 ± SD 10.81]	40–49	5 (3.3)
[Range 34–89]	50–59	33 (21.9)
	60–69	50 (33.1)
	70–79	43 (28.5)
	80–89	17 (11.3)
Smoker	No	132 (87.4)
	Yes	19 (12.6)
Alcoholic	No	141 (93.4)
	Yes	10 (6.6)
Comorbidities	Hypertension	120 (79.5)
	Diabetes mellitus	58 (38.4)
	Stroke	29 (19.2)
	Hyperlipidemia	24 (15.9)
	Congestive heart failure	23 (15.2)
	Ischemic heart disease	20 (13.2)
	Chronic kidney disease^∗^	10 (6.6)
	Hyperthyroidism	7 (4.6)
	Gout	3 (2.0)
	Valvular heart disease	3 (2.0)


*^∗^Only one case for end-stage renal failure was found.*

**Table 2:** Risk scoring of included patients.

Risk score	Score	*n* (%)
**CHA2DS2VASc**		
[Median = 3, mean 0.11 ± SD 1.40]	0	8 (5.3)
[Range 0–6]	1	28 (18.5)
	2	37 (24.5)
	3	40 (26.5)
	4	26 (17.2)
	5	7 (4.6)
	6	5 (3.3)
**HASBLED score**		
[Median = 1, mean = 1.25 ± SD 0.93]	0	35 (23.2)
[Range 0–4]	1	59 (39.1)
	2	43 (28.5)
	3	13 (8.6)
	4	1 (0.7)


### INR and TTR Levels before and after Starting Protocol

Differences in INR and TTR levels between pre- and post-protocol groups were tested using Mann–Whitney test. The TTR level was higher in WMTAC group, but was not statistically significant (**Table 3**). To facilitate comparison between interventional groups, these patients’ results in terms of TTR were divided into five groups (**Table 4**).

**Table 3:** International normalized ratio (INR) and time in therapeutic range (TTR) in UMC and WMTAC.

	UMC (*n* = 126)	WMTAC (*n* = 106)	*p*-value
INR level, mean ± SD	2.09 ± 0.31	2.18 ± 0.30	0.04^∗^
TTR level, mean ± SD	59.25 ± 20.74	63.97 ± 19.41	0.12


*^∗^*p*-value is significant < 0.05.*

**Table 4:** Time in therapeutic range (TTR).

TTR reading (%)	UMC	WMTAC
	(*n* = 126, %)	(*n* = 106, %)
**Range 1–100**		
1–20	17 (11.2)	15 (9.9)
21–40	33 (21.9)	39 (19.2)
41–60	51 (33.8)	42 (27.9)
61–80	53 (35.1)	64 (42.3)
81–100	33 (21.9)	59 (39.1)


### Pharmacist’s Recommendation Accepted, Expanded Therapeutic INR Range and Missed Doses Events

Results revealed that the pharmacists’ involvement in the WMTAC clinic had a positive impact, as more pharmacist recommendations were accepted in the WMTAC than UMC group (*p* = 0.01; **Table 5**).

**Table 5:** Effect of UMC versus WMTAC on anticoagulation control.

Interventions		UMC	WMTAC
		(*n* = 126, %)	(*n* = 106, %)
Accepted pharmacist recommendation	Yes	119 (94.4)	106 (100.0)
	No	7 (5.6)	0 (0.0)
Expanded therapeutic INR range	Yes	98 (77.8)	96 (90.6)
	No	28 (22.2)	10 (9.4)
Missed doses	Yes	0 (0.0)	1 (0.9)
	No	126 (100.0)	105 (99.1)


## Discussion

### Bleeding Complication

Most of the patients did not experience bleeding symptoms either before or after the implementation of the protocol (**Table 6**). This may be due to interventions and proper counseling during counseling sessions with patients. All symptoms such as bleeding or thrombotic symptoms were included in the counseling part and charted in the progress notes of the patients. A study conducted by Rossiter et al. (2013) stated that patients on warfarin treatment can be associated with hemorrhagic side-effects. For example, they may be prone to gastrointestinal bleeding and hemorrhagic strokes (Rosendaal et al., 1993). A study by Poon et al. (2007) illustrated that minor bleeding events were more frequent among the pharmacist-monitored group with 50 patients against 17 patients in UMC, with *p* < 0.01. This study adopted a standardized electronic template that specifically listed each minor bleeding symptom, where the pharmacists reviewed and documented symptoms with the patients during each anticoagulation service encounter (Poon et al., 2007). The findings from the current study were consistent with the findings of the study by Poon et al. (2007) as more bleeding events were detected during the sessions with the WMTAC group.

**Table 6:** Reported bleeding events of included patients.

Types of bleeding	UMC	*n* (%)	WMTAC	*n* (%)
Number of events bruises	No bleeding event	143 (94.7)	No bleeding event	125 (82.8)
	One event	4 (2.6)	One event	20 (13.2)
	Two events	4 (2.6)	Two events	5 (3.3)
	Three events	0 (0.0)	Three events	0 (0.0)
	Four events	0 (0.0)	Four events	1 (0.7)
Gum bleeding	No bleeding event	146 (96.7)	No bleeding event	139 (92.1)
	One event	4 (2.6)	One event	8 (5.3)
	Two events	0 (0.0)	Two events	4 (2.6)
	Three events	1 (0.7)	Three events	0 (0.0)
Blood in saliva	No bleeding event	150 (99.3)	No bleeding event	149 (98.7)
	One event	0 (0.0)	One event	1 (0.7)
	Two events	1 (0.7)	Two events	1 (0.7)
Hematuria	No bleeding event	150 (99.3)	No bleeding event	143 (94.7)
	One event	1 (0.7)	One event	7 (4.6)
	Two events	0 (0.0)	Two events	1 (0.7)
Blood in stools	No bleeding event	150 (99.3)	No bleeding event	140 (92.7)
	One event	1 (0.7)	One event	11 (7.3)
	Two events	0 (0.0)	Two events	0 (0.0)
Nose bleed	No bleeding event	150 (99.3)	No bleeding event	149 (98.7)
	One event	1 (0.7)	One event	2 (1.3)
	Two events	0 (0.0)	Two events	0 (0.0)
Hemoptysis	No bleeding event	150 (99.3)	No bleeding event	147 (97.4)
	One event	1 (0.7)	One event	3 (2.0)
	Two events	0 (0.0)	Two events	1 (0.7)
Hematemesis	No bleeding event	151 (100)	No bleeding event	150 (99.3)
	One event	0 (0.0)	One event	1 (0.7)
Hematoma	No bleeding event	150 (99.3)	No bleeding event	149 (98.7)
	One event	1 (0.7)	One event	1 (0.7)
	Two events	0 (0.0)	Two events	1 (0.7)
Blood in phlegm or sputum	No bleeding event	147 (97.4)	No bleeding event	147 (97.4)
	One event	4 (2.6)	One event	4 (2.6)
Subconjunctival hemorrhage	No bleeding event	150 (99.3)	No bleeding event	148 (98.0)
	One event	1 (0.7)	One event	3 (2.0)
	Two events	0 (0.0)	Two events	0 (0.0)
Cerebrovascular accident (CVA)	No bleeding event	151 (100)	No bleeding event	149 (98.7)
	One event	0 (0.0)	One event	1 (0.7)


A study by Jennings et al. (2008) from 2004 to 2006 showed that a pharmacist’s involvement in anticoagulation management can lead to a reduction in thromboses (4.6 to 3.9%), major bleeds (8.7 to 3.3%), and minor bleeds (4.6 to 3.5%). Hence, pharmacists do have important roles in managing warfarin therapy according to the protocol.

### INR and TTR Readings in UMC and WMTAC Group

INR levels were analyzed in these two groups: UMC and WMTAC. A similar comparison was done by Saokaew et al. (2013), who reported a significant improvement in INR level from the UMC to the WMTAC group. When compared to UMC group, implementation of pharmacist-managed warfarin therapy (PMWT) significantly improved patients’ anticoagulation control (Saokaew et al., 2013). Similar findings were found in a study by Chamberlain et al. (2001) where the anticoagulation clinic group had better anticoagulation control than the traditional care group. Comparing INR values outside the target range produced significantly more variation within the traditional care group (Chamberlain et al., 2001).

A study by Poon et al. (2007) indicated that pharmacist-monitored anticoagulation therapy significantly reduced thromboembolic events among older patients compared to the traditionally monitored groups. The study also reported that more patients in the traditional physician-monitored group had sub-therapeutic INR measurement ≤1.5 and they did not return as quickly for dosage adjustment (Poon et al., 2007). In addition, a prospective study by Padron and Miyares (2013) carried out in the Jackson Memorial Hospital, Miami, reported that involvement of pharmacists in anticoagulation management can enhance standards of care and ensure the protocols were upheld, therefore improving patients’ outcomes.

Results from a cross-sectional study by Hasan et al. (2011) found that MTAC patients showed better INR control (*p* = 0.006) than patients from the physician clinic. The study also showed a positive correlation between patients’ knowledge and education level (*p* = 0.001; Hasan et al., 2011). While physicians in cardiology clinics were responsible for both AF patients and patients with other cardiovascular issues, MTAC pharmacists concentrated on warfarin patients only. The study asserted that such focus of pharmacists helped to improve patients’ INR control (Hasan et al., 2011).

In current study, although INR level of WMTAC group is significantly higher than UMC group (**Table 3**), the reported values were both within targeted INR range. However, patients’ education level was not evaluated in current study. There was also no assessment of the adherence of patients, as this study was a retrospective study.

In this study, the TTR levels were compared between these two groups: UMC and WMTAC. The results show an improvement in TTR group 61–80 increasing from 35.1 (*n* = 53) to 42.3% (*n* = 64). In addition, TTR group 81–100 also showed a positive improvement as the TTR level improved from 21.9 (*n* = 33) to 39.1% (*n* = 59). This result indicates positive impact of pharmacist-managed WMTAC clinic (see **Table 5**).

Connolly et al. (2008) reported that a minimum TTR of 60% is vital to achieve treatment benefit of warfarin. Patients who failed to achieve such target were documented to have significantly greater risk of myocardial infarction, stroke and systemic embolism event (White et al., 2007). Hence, the findings of current study show the benefits of pharmacists’ interventions in warfarin therapy.

Young et al. (2011) stated that both models of care (UMC and pharmacist-led) may provide high-quality warfarin management, resulting in TTR of over 60% in both groups. Nonetheless, the results indicated that the patients from pharmacist care group have significantly greater TTR (73%) than UMC group (65%). The study also documented higher expanded TTR in pharmacist-led group (*p* < 0.0001). The warfarin therapy from this study was managed by delivering optimization of anticoagulation therapy using an evidence-based protocol developed by pharmacists (Young et al., 2011).

Meanwhile, studies done previously (Rosendaal et al., 1993; Witt et al., 2005; Young et al., 2011) have suggested that coordinated care and systemic approach in anticoagulation management lead to improved outcomes and reduced adverse events. Point-of-care INR clinic by pharmacists has also been reported to increase TTR significantly among warfarin patients (Rossiter et al., 2013).

According to a study undertaken in the Singapore General Hospital by Kong et al. (2012), there was a superior enhancement in the serial percentage of TTR (from 44.78 to 54.44% with *p* < 0.05) when pharmacist-led care was introduced. Overall findings showed that a low number of thrombotic and bleeding events were detected. However, no statistical significant differences were found during the study periods. In this study, the delivery of anticoagulation care in the clinics necessitate the services of well-trained pharmacists in order to ensure a greater consistency, as pharmacists undertake a more substantial amount of the workload. This study also documented a correlation between increased number of experienced pharmacists and patients’ TTR level (Kong et al., 2012). However, due to retrospective nature of current study, the number and skill of WMTAC pharmacists could not be determined.

### Pharmacist’s Recommendation Accepted, Expanded Therapeutic INR Range, and Missed Doses Events Interventions

The results show that expanded therapeutic INR range was statistically significant with a *p*-value of 0.04. According to the WMTAC protocol, an expanded INR reading within ±0.2 of the actual reading can be accepted without any complications (**Table 5**). Similar expanded range has been used in other studies (Jo-Anne Wilson et al., 2003; Lalonde et al., 2008).

In this study, a few interventions were included during consultation with patients during WMTAC, for example, pharmacists’ recommendation in terms of dosage adjustment, expanded therapeutic INR range, and missed doses for the past week detected during WMTAC. Pharmacists also counsel and monitor on drug–drug interactions, food interactions, and consumption of traditional medicine or supplements. Patients are educated with a list of green vegetables to eat and a list of several common traditional medicines or supplements. The results show the positive impact of pharmacists’ recommendations in the WMTAC, as the number of pharmacist recommendations accepted by the physician was significantly increased in the WMTAC compared to the UMC group. The pharmacists involved in WMTAC use the warfarin protocol and have undergone training to learn about dosage adjustment. Adjustment is done on the spot after the patients’ INR is detected. Hence this method also reduces patients’ waiting time in the clinic.

Gupta et al. (2013) illustrated that the clinical pharmacists exhibit their roles through assessment of patients’ adherence and understanding of warfarin therapy, and frequent monitoring of INR levels. Furthermore, pharmacists often detect and manage adverse events or other medication-related problems. According to the WMTAC protocol, all these details are necessary for the purposes of counseling and charting progress. Pharmacists will intervene and counsel toward increasing compliance and adherence to warfarin therapy, as well as clinically significant changes in diet (Gupta et al., 2013).

Findings of the study by Rossiter et al. (2013) showed that in family medicine settings, pharmacist-led INR clinic improved control of anticoagulation therapy among AF patients on warfarin therapy. There were similar findings in a study by Poon et al. (2007) where the pharmacist-monitored anticoagulation services were documented to lead to favorable outcomes in both efficacy and warfarin-related complications. However, this study only focused on patients over 75 years old.

After implementation of WMTAC protocol, greater emphasis was given on pharmacists’ counseling on the compliance issue, and to educate patients regarding missed doses and time adjustment. Nevertheless, in current study, there was no significant difference in missed doses among UMC and WMTAC groups, as only one missed dose event was recorded throughout the study.

### Strengths and Limitations of the Study

This study adopted an observational methodology with a retrospective cohort design. A retrospective design was deemed appropriate as the data utilized in this study was already recorded in a systematic way as part of patient care and the medical staff were expected to routinely complete data documentation. Generally, observational studies represent a valuable component in investigation of treatment outcomes, and offer balanced evidence base for clinical decision-making (Ligthelm et al., 2007). Such detailed observations can be reliably replicated and generalized to similar conditions or populations.

Retrospective data recalls exposures occurring sometime in the past and are collected by searching medical records (Luepker, 2005). In addition, medical records are reliable data sources for evaluation of existing conditions (Thomas et al., 2002).

Retrospective studies rely on medical staff documentation rather than patient interviews. In the current study, all variables needed to evaluate the objectives could be obtained from medical staff and pharmacists’ documents, as well as information sometimes obtained from the caregivers instead of the patients themselves.

To minimize the potential for missing data, current study measured confounding variable for all probable relevant variables. All warfarin patients entered into the study were followed up for the duration of the study. To avoid the effect of data losses on validity of the results, missing data was minimized by using prediction of regression statistics.

Moreover, retrospective data can be free from researcher bias. In this study, all patients fulfilling the inclusion criteria were screened, and researcher collected the relevant data with no preconceived views of possible findings. Furthermore, during review of the medical charts, a review was conducted of the notes of cardiology consultants and internal medical physicians, who are required to document the full history, diagnosis, management, recommendations, and preventive care strategies for all patients. Besides that, usage of retrospective medical chart review is cheaper and quicker to complete, while maintaining reliability of information (Thomas et al., 2002). Data collection is reassessed and reviewed by other researchers to reduce research bias.

As this observational study was conducted in one hospital only, the study has limitations in scope and generalizability. Furthermore, due to limited sample size, the study did not involve patients with other ailments (such as pulmonary embolism and deep vein thrombosis) who were also attending warfarin clinic.

Randomization of patients was not considered, as this study involved various modalities of treatment across the whole unit, and a matched control unit was not available.

## Conclusion

There is a significant positive association between the pharmacist-led WMTAC and anticoagulation effect (therapeutic TTR, INR). The identified findings show that expanded role of pharmacist in PMWT is beneficial to optimize the warfarin therapy. In such therapy, recommendations are made such as dose adjustment, counseling on adherence and safer alternative drugs (given drug–drug interactions and/or food–drug interactions). This study also highlights the critical role that pharmacists can actively play to ensure optimal anticoagulation pharmaceutical care in collaboration with other healthcare teams.

## Author Contributions

SA, TK, CN, SS, and LM conceived the concept. SA, YS, CY, TK, CN, SS, and LM wrote the initial draft. SA, YS, CY, CN, YK, RP, and LM finalized the manuscript. All authors contributed toward revising the paper and agree to be accountable for all aspects of the work.

## Conflict of Interest Statement

The authors declare that the research was conducted in the absence of any commercial or financial relationships that could be construed as a potential conflict of interest.
